# Simultaneous presentation of dual pathologies within the oral cavity: an unusual and diagnostically challenging presentation

**DOI:** 10.1038/s41415-023-6138-5

**Published:** 2023-08-25

**Authors:** Beant Singh Thandi, Rosemarie Jane, Vinay Chavda, Iain L. C. Chapple

**Affiliations:** 510156266965599298450 School of Dentistry, University of Birmingham, Birmingham, United Kingdom; 449531217429967310735 School of Dentistry, University of Birmingham, Birmingham, UK; Periodontal Research Group, Institute of Clinical Sciences, College of Medical and Dental Sciences, University of Birmingham, United Kingdom

## Abstract

Necrotising sialometaplasia (NS) is a rare condition, with a limited scientific evidence base regarding its aetiology and pathophysiology. Diagnosing NS demands extensive investigatory tests. Their accuracy is vital in order to exclude oral malignancy and prevent unwarranted, invasive management.

Within Birmingham Dental Hospital, a 22-year-old, South Asian woman presented with generalised pain from the lower right third molar extending to involve the palate, to which the patient's general medical practitioner previously attributed to a viral upper respiratory infection. Clinical examination revealed bilateral erythematous: non-ulcerated, palatal swellings (10 mm x 5 mm) at the greater palatine foramina. Following extensive investigations, the challenging definitive diagnoses of two distinct pathologies were made: non-ulcerative NS and pericoronitis.

This case report describes the successful diagnosis and management of non-ulcerating NS, an 'atypical' presentation of a rare condition, that was confounded by a simultaneous episode of pericoronitis - a presentation not previously documented within scientific literature.

## Key points

- Readers will appreciate the importance of investigating suspicious lesions: if in doubt - refer!
- Recognise the importance of listening to patient complaints, particularly when handling multiple complaints.
- Develop an appreciation of the special tests/investigations required in reaching a diagnosis of necrotising sialometaplasia.

## Clinical presentation

A 22-year-old, South Asian woman, who was otherwise fit and well, presented to the urgent care department of Birmingham Dental Hospital. The patient complained of a two-day history of severe pain associated with her lower right and left third molar regions and a severe burning pain associated with the roof of her mouth. The pain began as a sore throat with a mild fever and was diagnosed as a viral upper respiratory infection by her general medical practitioner, who advised symptomatic relief (analgesics and anti-pyrexials) alongside rest. However, following its sudden onset, the pain worsened, causing sleep disturbance and restricting her diet to soft foods, leading to her seeking further advice. There was no prior history of such pain, nor of recent intra-oral trauma.

Extra-oral examination revealed bilateral submandibular lymphadenopathy, mild trismus and right masseteric tenderness.

**Fig. 1:**
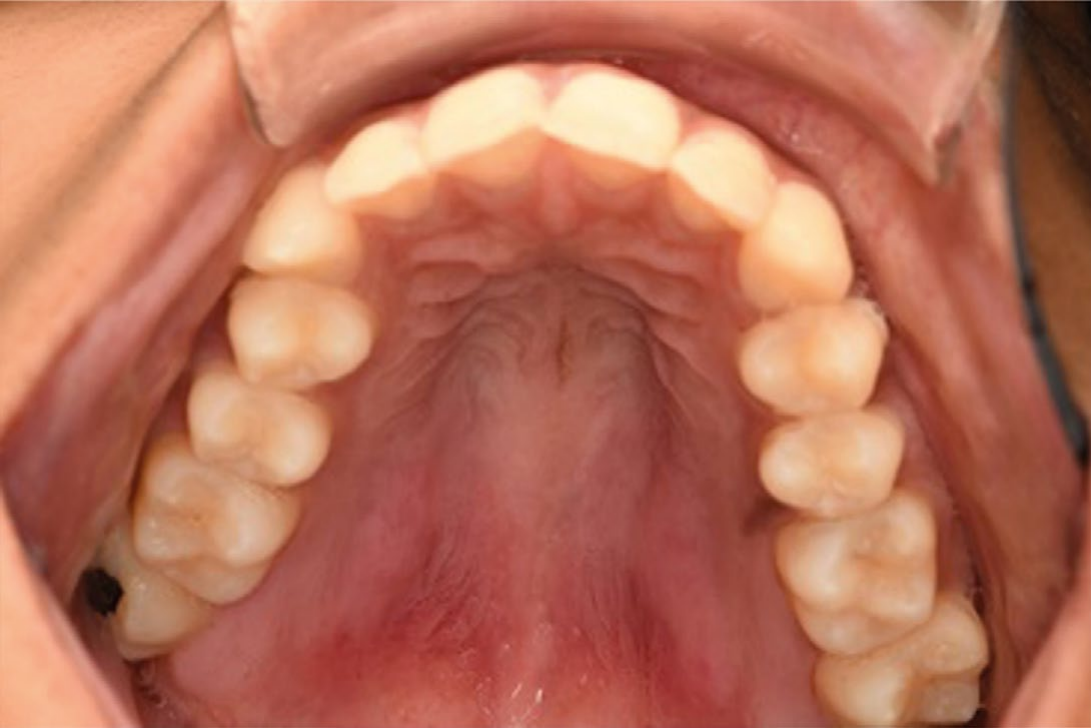
Bilateral swelling over the greater palatine vessels of the palate, no necrotic tissue visible

**Fig. 2:**
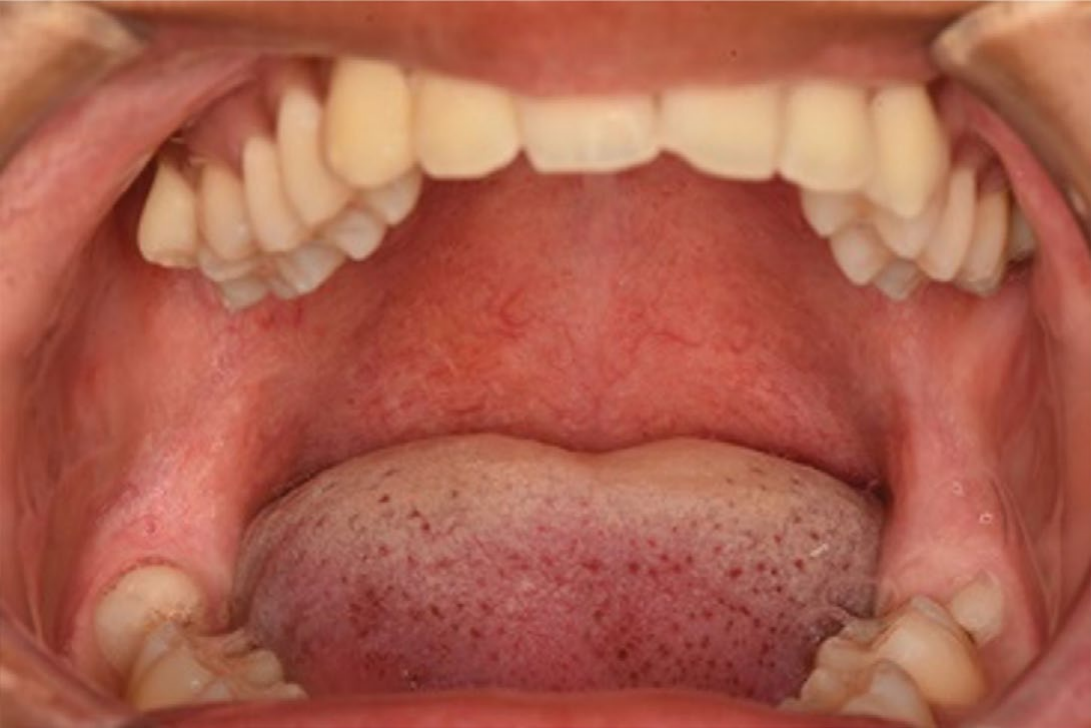
Partially erupted lower left and right third molar, mild visual signs of inflammation

Intra-oral examination revealed bilateral swellings on the hard palate in the region of the greater palatine foramina, both of which were mildly tender on palpation. Both swellings were 10 mm in length antero-posteriorly, and 5 mm in width medial-laterally (Fig. 1). Both lower third molars were partially erupted, displaying mild clinical signs of inflammation affecting the distal soft tissue operculum (Fig. 2).

Special investigations undertaken included a dental panoramic tomogram (DPT), blood samples for full blood count, serum immunological analysis (including immunoglobulin G and M isotypes, complement C3 and C4, and К and λ free light chain quantities and ratio). Clinical photographs were also taken for monitoring purposes to determine if the lesions were developing or reducing in size (Fig. 1, Fig. 2). An ultrasound scan was scheduled to determine the vascular nature of the palatal lesions.

**Fig. 3:**
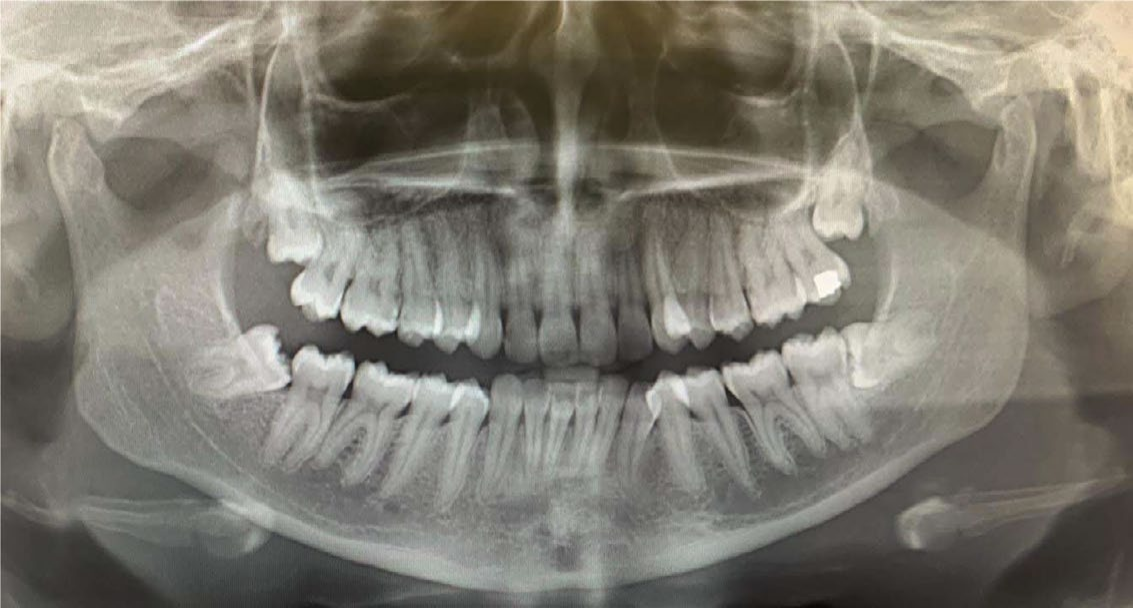
Dental panoramic tomogram revealed mesio-angular impaction of the lower right wisdom tooth and horizontal impaction of the lower left wisdom tooth against their respective lower second molars

Within the initial visit, the DPT revealed mesio-angular impaction of the lower right third molar and horizontal impaction of the lower left third molar against their respective lower second molars (Fig. 3). A definitive diagnosis of bilateral pericoronitis associated with both lower third molars with no systemic involvement was reached.

This was managed with debridement of the lower third molars and irrigation of the operculum with chlorhexidine digluconate (Corsodyl 0.2% mouthwash, GlaxoSmithKline). The patient was advised to maintain good oral hygiene, perform chlorhexidine digluconate mouth rinses three to four times daily for a week, and to rest. Ibuprofen was recommended as an analgesic to manage the pain associated with the acute pericoronitis and the palatal lesions of unknown aetiology.

### Differential diagnoses

Prior to the results of the remaining special investigations, the differential diagnoses of the bilateral palatal lesions were:

- Bilateral pleomorphic adenomas
- Extranodal non-Hodgkin lymphoma or lymphoproliferative disease
- Bilateral necrotising sialometaplasia (NSs)
- Mucoepidermoid carcinomas
- Squamous cell carcinomas (SCCs).

### Special investigation results

**Fig. 4:**
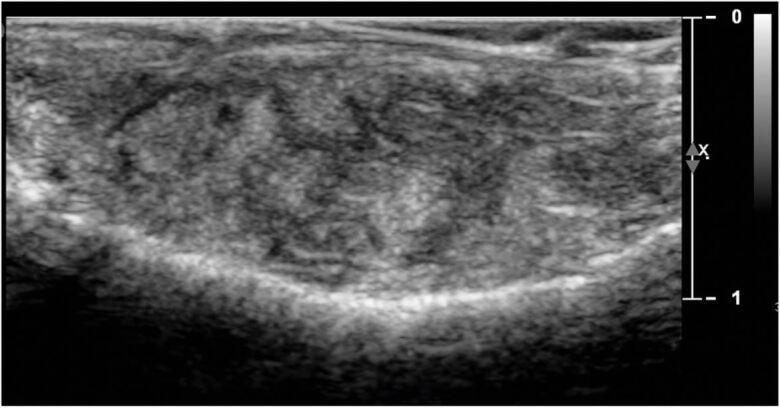
Ultrasound of the palatal swelling. A solid tissue mass with minimal vascularity and no fluid collections or calcifications

**Fig. 5:**
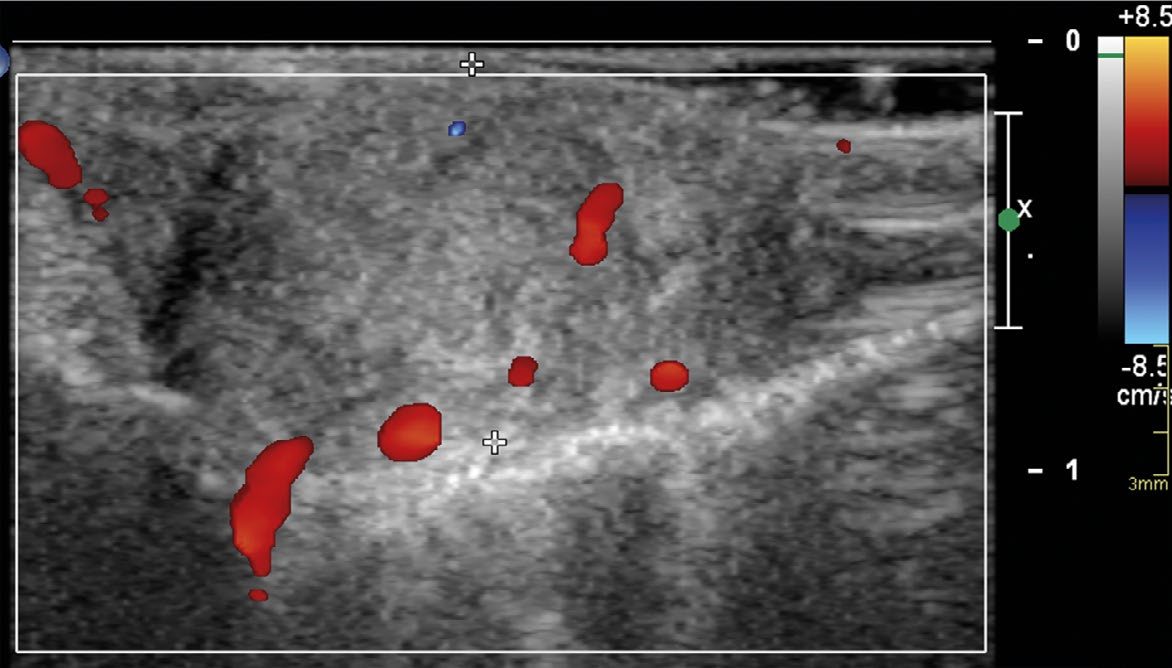
Areas of minimal vascularity outlined on palatal swelling

The full blood count and serology were all within normal ranges (Table 1, Table 2). The ultrasound scan revealed solid tissue on the affected palatal areas, measuring up to 9 mm in thickness, with minimal vascularity and no calcification, or fluid collection (Fig. 4, Fig. 5).

**Table 1:** Full blood count

Full blood count	Quantities	Average range
Haemoglobin	135 g/L	130-180 g/L
Platelets	363 x 10/L^9^	140-400 x 10/L^9^
Red blood cell	4.58 x 10/L^12^	3.80-5.80 x 10/L^12^
Neutrophils	4.8 x 10/L^9^	1.8-7.5 x 10/L^9^
Eosinophils	0.12 x 10/^9^	0.1-0.4 x 10/L^9^

**Table 2:** Serology results

Serology	Quantities	Average range
Immunoglobin isotype G	12.44 g/L	6.0-16.0 g/L
Immunoglobin isotype M	0.68 g/L	0.4-2.5 g/L
Complement C3	133 mg/dL	90-180 mg/dL
Complement C4	32 mg/dL	20-50 mg/dL
Kappa free light chain	13.01 mg/L	3.3-19.4 mg/L
Lambda free light chain	15.12 mg/L	5.71-26.3 mg/L
Kappa/lambda free light chain ratio	0.86	0.26-1.65

**Fig. 6:**
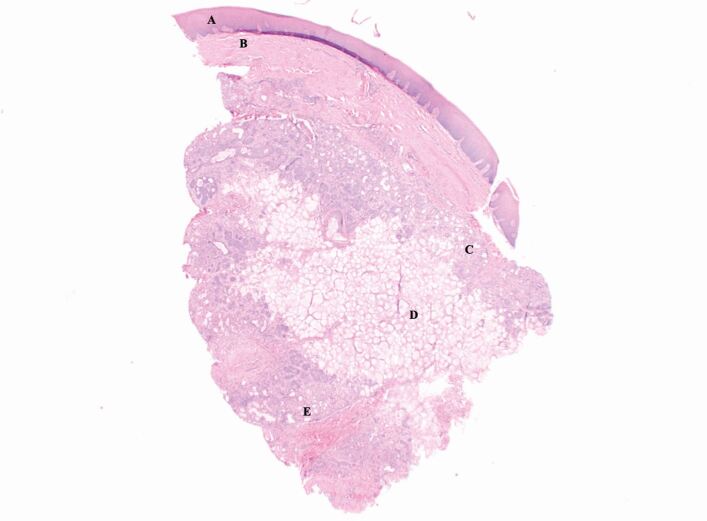
Incisional biopsy of the left hard palate (greater palatine region). A = squamous epithelium; B = lamina propria; C = peripheral zone displaying acinar atrophy and squamous metaplasia; D = central zone of coagulative necrosis; E = peripheral zone of acinar atrophy and squamous metaplasia

**Fig. 7:**
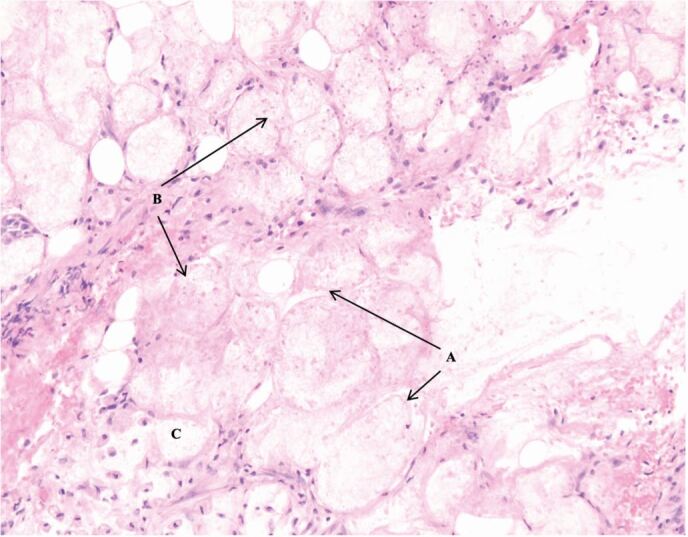
Central zone of coagulative necrosis. A = necrotic mucous acini retaining cellular outlines; B = residual nuclear material, pale in appearance and either shrunken or fragmented; C = cytoplasm pale and clumped

**Fig. 8:**
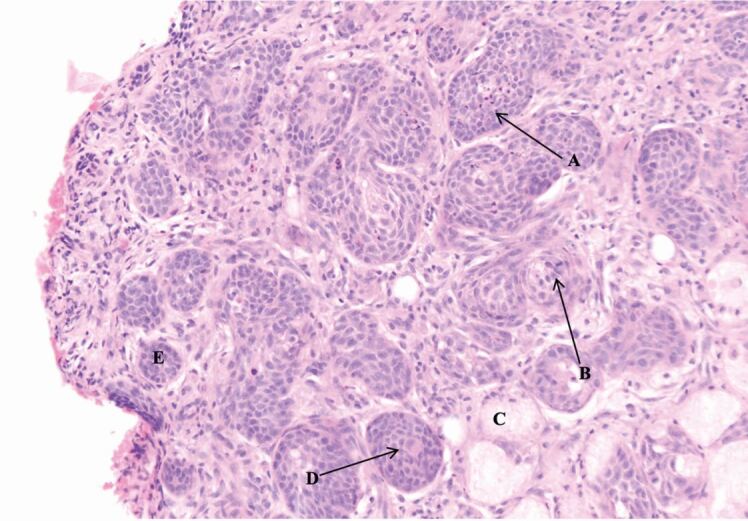
Peripheral zone displaying squamous metaplasia. A = apoptotic debris; B = mitotic figure; C = admixed mucous cells; D = early keratinisation towards the centre of lobules; E = lobules of squamoid cells with smooth, rounded outlines

**Fig. 9:**
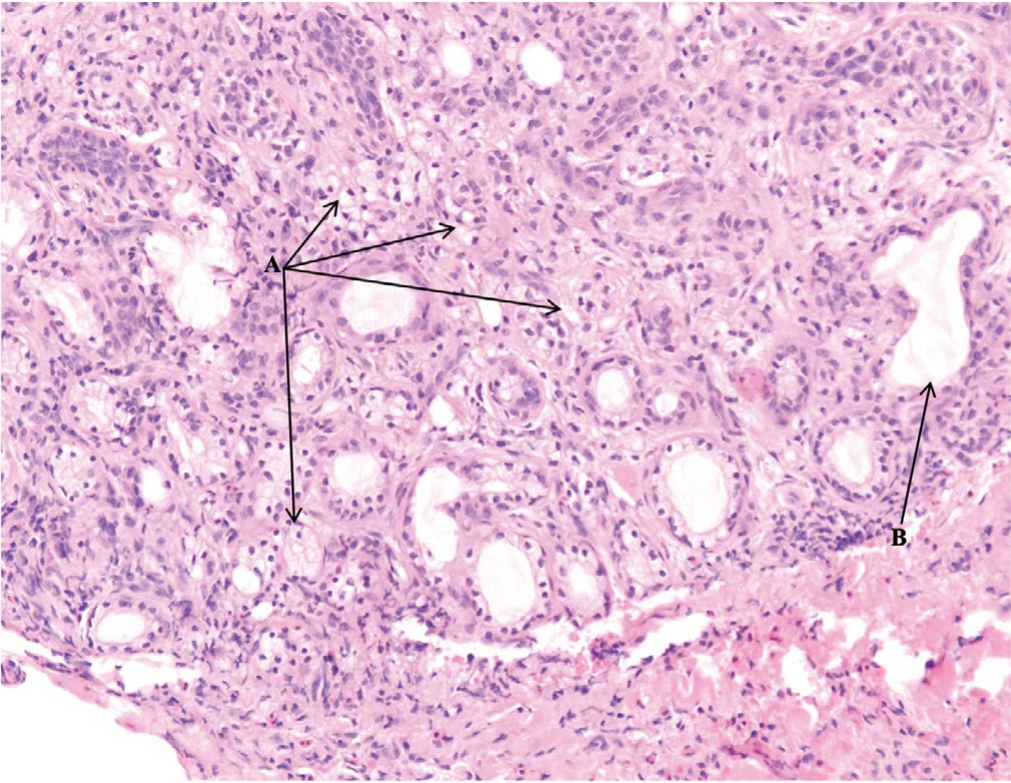
Peripheral zone, atrophic changes. A = atrophic mucous acini resembling clear cells; B = distended duct lined by flattened cells (note: no squamous metaplasia observed at time of biopsy within this duct)

At review three weeks later, the palatal lesions had remained tender to palpation, with the above special investigations solely excluding the diagnosis of extra-nodal non-Hodgkin lymphoma or lymphoproliferative disease. Thus, an incisional biopsy was undertaken and sent for histopathological analysis. This demonstrated acinar atrophy and squamous metaplasia and a central zone of coagulative necrosis (Fig. 6, Fig. 7, Fig. 8, Fig. 9). The lesion was confirmed as benign due to the biopsy's overall cellular and tissue architecture, specifically the smooth, consistent outline of the squamous lobules.

### Diagnosis

A definitive diagnosis of non-ulcerated, bilateral NS of the hard palate was reached.

### Management

The patient was educated about the condition and reassured that it was self-limiting and likely to resolve within three months. Symptomatic advice was reinforced. During the SARS-COV-2 pandemic, patient follow-up was via telephone consultation. Complete resolution of symptoms was reported at three months. The patient subsequently failed to attend further appointments for face-to-face review and was discharged back to primary care.

## Discussion

NS is a benign inflammatory lesion affecting the mucoserous glands of the palate, accounting for 0.03% of all lesions biopsied from the oral cavity.^1^^,^^2^ The aetiology of NS is postulated to be trauma-induced ischaemia of the mucoserous glands. Examples of such trauma include dental injections, ill-fitting dentures, adjacent tumours, sites of previous surgery and alcohol consumption, among others.^3^ NS should not be excluded when trauma is not recalled by the patient, as many cases of NS, including this case, have no reported prior traumatic events.

The average age of patients diagnosed with NS is 45.5 years old, with a slight predilection for men (1.76:1) and those of European descent. NS presents most commonly on the posterior hard palate as a painful ulcer that extends to expose underlying bone, occasionally preceded by a non-ulcerated swelling. In approximately one-third of cases, NS presents as a non-ulcerated swelling. NS has been identified in other sites within the head and neck, including the lower lip, tongue, buccal mucosa, major salivary glands (frequently the parotid gland), nasal cavity, maxillary sinus and larynx. The average diameter of NS has been reported as 18 mm, ranging from 7-50 mm.^3^ Brannon *et al.*, reported NS lesions of the palate (n = 36) to present more frequently unilaterally (88.9%) than bilaterally (2.8%).^3^ The clinical and histopathological features of NS mimic those of malignant neoplasms.^4^ Thus, reaching the correct definitive diagnosis is of the utmost importance to prevent the patient undergoing unnecessary invasive treatment and emotional turmoil that can result from an incorrect diagnosis.^1^^,^^3^ Management of NS remains primarily centred around symptomatic relief, patient education and reassurance, and monitoring for spontaneous healing.^5^

Clinical features of NS include a non-healing ulcer with indurated margins, which is typically tender and visibly sore, giving the impression of an oral malignancy, such as an SCC. In the current case, the presentation was rather unique in that the lesions were non-ulcerative in nature, a potential diagnostic pitfall given the rarity of NS. A literature review of the PubMed database utilising the following search terms and Boolean operators - 'necrotising sialometaplasia' AND 'non-ulcerated' AND/OR 'non-necrotic' - revealed 16 reported cases of non-ulcerative NS from 2000-2021 written within the English language.^6^^,^^7^

This slow-growing, firm mass presenting within the palate with a normal appearance of the overlying mucosa indicated the possible diagnosis of bilateral pleomorphic adenomas (PAs). PAs are the most common salivary gland tumour, most frequently identified within the palate in women within the fourth to fifth decades of life; however, they may occur at any age. PAs are benign mixed tumours of epithelial and myoepithelial cells and, as found within this case, induce limited (if any) bony resorption, and one consistent with the ultrasound imaging report of a solid tissue mass with minimal vascularity and no fluid collections or calcifications. The absence of pain associated with the patient's lesions and the bilateral presentation, in addition to the histopathological report demonstrating a lack of myoepithelial cells, helped to eliminate PAs from the differential diagnosis.^8^

Non-Hodgkin's lymphoma is the second most common malignancy of the head and neck, following SCC. Extranodal non-Hodgkin lymphoma, such as diffuse large B cell lymphomas, frequently present within the palate as ulcerative swellings.^9^ Therefore, haematological and serological analysis was undertaken to eliminate lymphoproliferative conditions from the differential diagnosis, as it was thought that the swellings had been examined before their spontaneous ulceration. When investigating for B cell lymphomas, kappa and lambda free light chain quantities were analysed, as these are frequently elevated above the normal reference ranges in such conditions, in 82% and 92% of cases, respectively.^10^ Free light chains represent 'excess' light chains produced by plasma cells that are actively synthesising immunoglobulins but which are not utilised in immunoglobulin production themselves. Non-Hodgkin lymphoma was ruled out due to both haematological and serological results being within normal ranges.

There are features in the presented case that could have led to the misdiagnosis of a SCC or mucoepidermoid carcinoma. Within the histopathology report, the presence of mitotic figures, apoptotic debris and early keratin formation (Fig. 8)acted as false flags for SCC. Furthermore, the presence of mixed mucous, squamoid cells and clear cells (Fig. 9) suggested the diagnosis of a mucoepidermoid carcinoma.

It is common for the histopathology of the overlying palatal mucosa of NS to exhibit pseudoepitheliomatous hyperplasia, a feature often identified in SCCs and mucoepidermoid carcinomas;^11^ however, this was not reported in the histopathological examination. Although the histopathology report in this case was unable to identify all histopathological features of NS, the biopsy's overall cellular and tissue architecture, in particular the smooth and consistent outline of the squamous lobules, justified ruling out SCC and mucoepidermoid carcinoma from the differential diagnosis.

Characteristic histological features that make NS identifiable include lobular necrosis of salivary glands, squamous metaplasia of ducts and acini, mucus extravasation and inflammatory cell infiltration. The histopathology report demonstrated several such features, which, combined with the clinical examination and the results of the additional special investigations, led to the definitive diagnosis of NS. If any ambiguity remains over the diagnosis, immunohistochemistry analysis can be used to confirm findings. Haematoxylin and eosin staining resins, as used in this case report, remain the current gold standard for a histopathological diagnosis.^11^ Immunohistochemical findings to substantiate a diagnosis of NS include the demonstration of absent to focal (**≤** 5% of cells) immunoreactivity for the tumour protein p53, decreased immunoreactivity for E3 ubiquitin protein ligase 1, mindbomb 1 (Ki67) and the presence of 4A4/tumour protein p63 and calponin-positive myoepithelial cells.

The management of NS involves educating and reassuring the patient about the condition, explaining it will resolve without intervention in approximately three months and the recommendation of appropriate analgesics during this healing period. The clinician should review the patient for spontaneous healing and should there be any changes in clinical presentation or doubt over the diagnosis, further biopsy and histopathological analysis must be undertaken. In such cases where NS is most likely and malignancy a possible differential diagnosis, this conservative monitoring approach safeguards the patient from misdiagnosis and ensures unnecessary and aggressive surgical intervention is avoided.^1^

## Conclusion

This case report aimed to share the learnings from the successful diagnosis and management of this rare condition, albeit presenting in an 'atypical' form (non-ulcerated NS), confounded by a concomitant acute episode of bilateral pericoronitis - a presentation not previously documented within the scientific literature.

The chief complaint of the patient was associated with her lower third molars, attributing the palatal pain to a sore throat, which, without a thorough intra-oral soft tissue examination, the diagnosis of NS could have been easily missed due to its discrete, non-ulcerated presentation. Histologically, there was evidence to substantiate a diagnosis of non-ulcerative NS; however, key features, such as atrophic debris and early keratin production, acted as false flags for malignancy, making the case more challenging to accurately diagnose. NS is a rare condition; nevertheless, this case report demonstrated the need to review the patient to assess whether spontaneous healing occurred, to confirm the correct diagnosis of NS, and to undertake an additional biopsy if in doubt.
